# At the interface of asymptotics, conformal methods and analysis in general relativity

**DOI:** 10.1098/rsta.2023.0048

**Published:** 2024-03-04

**Authors:** G. Taujanskas, J. A. Valiente Kroon

**Affiliations:** ^1^ Department of Pure Mathematics and Mathematical Statistics, University of Cambridge, CB3 0WB, UK; ^2^ School of Mathematical Sciences, Queen Mary University of London, Mile End Road, London E1 4NS, UK

**Keywords:** null infinity, conformal compactification, general relativity, gravitational radiation, asymptotic structure of spacetime

## Abstract

This is an introductory article for the proceedings associated with the Royal Society Hooke discussion meeting of the same title which took place in London in May 2023. We review the history of Penrose’s conformal compactification, null infinity and a number of related fundamental developments in mathematical general relativity from the last 60 years.

This article is part of a discussion meeting issue ‘At the interface of asymptotics, conformal methods and analysis in general relativity’.

## 1. Introduction

Roger Penrose’s seminal idea [1,2] of using techniques from conformal geometry to study global questions in general relativity has been vastly influential. Today, these ideas permeate a number of current research programmes in mathematical physics, partial differential equations and analysis, and have contributed much to the basic language of general relativity from null infinity to Penrose diagrams. This article is an introductory article for the proceedings of a workshop of the same title, which took place 9–10th May 2023 at the Royal Society in London, aimed at providing a survey of the field 60 years after Penrose’s original work.

## 2. Penrose’s conformal compactification

Robert Geroch’s description in the proceedings [3] of the 1976 Cincinnati Symposium on Asymptotic Structure of Space-Time captures well an essential reason why general relativity (henceforth GR) is distinct from many other physical theories: it is that in GR, the notion of an ‘isolated system’ becomes much harder to define than in other theories. This makes GR significantly more difficult to study. For example, Newtonian gravitation is described by a potential ϕ evolving on fixed Euclidean 3-space, and a system may be said to be ‘isolated’ if the mass density vanishes outside some compact subset of this space, and ϕ decays to zero away from the compact set. In GR, however, such a separation of the fields into ‘background’ and ‘physical’ fields is much more difficult, as the spacetime metric ${\tilde{g}}_{ab}$ is both; here one has to construct the evolving field and its background at the same time. A natural attempt to define an isolated system in general relativity might be via an appropriate notion of ‘asymptotic flatness’ (at least in the case of zero cosmological constant Λ): as one recedes ‘to infinity’, the spacetime geometry should approach Minkowski space in a suitable sense, for example that the Riemann tensor of ${\tilde{g}}_{ab}$ should approach zero. However, this is mathematically awkward and somewhat non-geometric, since one is then faced with taking limits of tensor fields on manifolds, which is in general not well defined, as well as having to describe limits of differentiable structures.

### (a) Null infinity

Penrose’s idea of using a conformal compactification to address the problem described in the previous paragraph is particularly elegant since it in one stroke takes care of both of these difficulties. Consider the physical metric ${\tilde{g}}_{ab}$, and rescale it, $g_{ab}=\Omega^{2}{\tilde{g}}_{ab}$, using a conformal factor Ω, a smooth positive scalar field which approaches zero at infinity with respect to ${\tilde{g}}_{ab}$ at an appropriate rate. Penrose called $g_{ab}$ the *unphysical* or *rescaled* metric. If Ω is chosen correctly, the limit points of all inextendible null geodesics are then brought to a finite distance, and form a submanifold called *null infinity*, denoted I. Asymptotic considerations with respect to ${\tilde{g}}_{ab}$ are then replaced with local differential geometry in terms of Ω and $g_{ab}$ in a neighbourhood of I, and decay rates in null directions with respect to ${\tilde{g}}_{ab}$ may be reinterpreted in terms of regularity at I with respect to $g_{ab}$.

The massless fields in the physical spacetime, appropriately rescaled, then imbue I with information which may be thought of as a summary of the asymptotics of the processes in the physical spacetime. Conceptually, if one then detaches I from the rescaled spacetime and considers it, together with this summary of asymptotics, an object in its own right, the fields on I are found to split into two distinct classes, the geometrical, or *universal* fields, and the physical fields. The universal fields turn out to be, at least locally, the same no matter the original spacetime, and therefore deserve to be called universal. The physical fields, on the other hand, depend on the physical processes in the original spacetime. Penrose’s proposal therefore elegantly decouples the geometry from the physics, at least asymptotically, and for massless fields provides a way of defining an isolated system by requiring that a suitable compactification with Ω as described above exists.

### (b) Geroch conjectures from Cincinnati symposium

In [3] Geroch made three conjectures, numbered Conjecture 1, Conjecture 10 and Conjecture 13 (they were interspersed with Theorems). We mention them in reverse order. Conjecture 13 concerned the uniqueness of conformal completions of initial data sets for GR. That is, consider an asymptotically Euclidean 3-manifold $\tilde{T}$ (for simplicity, with just one asymptotic end) with a smooth positive definite metric ${\tilde{q}}_{ab}$ and a smooth symmetric tensor field ${\tilde{p}}_{ab}$, the extrinsic curvature. Consider a conformal completion of $\left(\tilde{T},{\tilde{q}}_{ab},{\tilde{p}}_{ab}\right)$ by a point S, $\left(T,q_{ab},p_{ab})=(\tilde{T}\cup S,\Omega^{2}{\tilde{q}}_{ab},\Omega^{-1}{\tilde{p}}_{ab}\right)$, where at S one has Ω=0, $\nabla_{a}\Omega =0$ and $\nabla_{a}\nabla_{b}\Omega =2q_{ab}$. Geroch called such a pair (T,Ω) an asymptote of $\tilde{T}$. Conjecture 13 asked whether any two asymptotes (T,Ω) and $\left(T^{\prime },\Omega^{\prime }\right)$ were *equivalent* in the sense that there exists a conformal rescaling ω such that ω=1 at S and $\left(T^{\prime },\omega \Omega^{\prime })=(T,\Omega \right)$. This conjecture has been essentially understood through an adaptation of the method of punctures to solve the constraint equations in GR [4–6], however, a proof does not appear to be explicitly present in the literature. We mention here also the earlier work of Ashtekar–Hansen, and their notion of AEFANSI (asymptotically empty and flat at null and spatial infinity) spacetimes [7], and the resulting notion of the Spi (spatial infinity) 4-manifold.

The second of Geroch’s conjectures, Conjecture 10, asked whether in a spacetime admitting a regular null I the vanishing of the (massless) Klein–Gordon or Maxwell radiation field at I implies that the solution should vanish in the interior of the spacetime. This is now known to be true in some generality as a consequence of the scattering theories constructed in [8–11] and elsewhere.

Finally, Conjecture 1 asked roughly whether, in a vacuum spacetime with a null I, linear perturbations of Einstein’s equations with compactly supported data (on a spacelike, perhaps hyperboloidal, hypersurface T) preserve I in the domain of dependence of T. In essence, this amounts to the question of the stability of spacetimes to linear gravitational perturbations (which, as posed by Geroch, should have compactly supported initial data). When the background is Minkowski space, this was answered affirmatively, in the full nonlinear regime, by Friedrich [12]. The celebrated work of Christodoulou & Klainerman [13] later proved the stability of Minkowski space for data on a uniformly spacelike Cauchy surface, doing away with Friedrich’s requirement that the data be prescribed on a hyperboloid (see also [14]); they obtained spacetimes with asymptotic behaviour which in general does not permit a smooth conformal compactification (see §2c for more on this). Even more general decay rates were later handled by Bieri [15] and more recently by Shen [16]. Back in the linear regime, recent work of Masaood [17,18] has handled the construction of a scattering theory for linear gravitational perturbations to a Schwarzschild black hole. A result of this kind for more general spacetimes possessing an appropriately regular null I appears to still be open. Related to this is the fact that the spacetimes admitting a regular null infinity are not fully characterized, although infinite-dimensional families of such spacetimes are known [19–22].

### (c) Peeling

The question of what ‘appropriate’ regularity of I should mean now arises naturally. In the late 1950s and early 1960s, Sachs [23,24] discovered (see also [25,26]) that in smooth asymptotically flat spacetimes a so-called peeling property was satisfied by the Weyl tensor. In essence, Sachs found that the different components of the Weyl tensor ‘peel off’ at decreasing, integer rate powers of the luminosity parameter r, $\Psi_{i}∼r^{i-5}$, 0≤i≤4 as one approaches null infinity along null directions. Peeling and the history of its discovery is described beautifully in the article of Penrose in these proceedings [27], and we refer the interested reader there. The important point, however, was the observation that the peeling property of the Weyl tensor is nothing more than a consequence of the smoothness of the Weyl tensor in the conformally compactified spacetime. This was a neat correspondence. However, for reasons that will be mentioned shortly, while the picture of an isolated system defined through a conformal compactification was appealing, it was not universally accepted by all workers in the field as appropriate. In particular, questions of generality, definability of relevant physical quantities, and the applicability of the model to physical situations of interest, remained—and still do. Calculations based on the equations of motion and the radiation escaping an isolated system to infinity did not always appear to verify the Sachs peeling property, which raised doubts as to the suitability of the smoothness of the compactified picture [28,29]. Indeed, there were concerns that the conformal picture did not admit *any* solutions to Einstein’s equations which contained gravitational radiation. This was put to rest by a series of results due to Corvino, Chruściel–Delay and Corvino–Schoen [19–22,30] (see also [31]), who showed that initial data for the Einstein equations could be deformed in more flexible ways than previously thought, e.g. by gluing essentially arbitrary small data in a compact region to Schwarzschildean asymptotics near spatial infinity. Friedrich’s semi-global stability result [12] with these data then produces an infinite-dimensional family of non-trivial solutions to the Einstein vacuum equations with complete and smooth conformal infinity I and regular past and future timelike infinities $i^{\pm }$. A more complete account of the history of peeling is given well by Friedrich in the review [32]. In general, if the decay of initial data is weaker than is required for peeling, even if the data is smooth towards spatial infinity $i^{0}$, Friedrich [33] has shown that the resulting evolution will in general be polyhomogeneous at I, a result of the degeneracy of Einstein’s equations at the points where spatial infinity meets null infinity. That quite generally the situation is no worse was recently confirmed by Hintz & Vasy [34]. Work to understand these issues sharply is ongoing, and the detailed structure of the polyhomogeneities exhibited by massless scalar fields on Minkowski space is reviewed in Gasperín’s [35] contribution to this issue.

The worry of mathematical genericity of peeling spacetimes being somewhat settled, the question of applicability to relevant physical situations nevertheless remains. At the time of writing, the general consensus appears to be that the smoothness assumptions in the classical definition of asymptotic simplicity are in some instances too restrictive to describe realistic physical scenarios. Kehrberger [36–38] has argued strongly that many systems of physical interest, e.g. the problem of N gravitating bodies coming in from infinity, violate not only peeling, but also the weaker decay assumptions and conclusions of Christodoulou–Klainerman [13,39]. There is some suggestion, however, that the weak assumptions of Bieri [15] are sufficient to capture these situations: see the chapter by Kehrberger [40] in this issue.

## 3. The BMS group

One of the main motivations behind Penrose’s approach to characterizing isolated systems in GR through conformal compactifications may have been that of identifying universal structures which, in turn, could be used as tools to study the physical processes taking place in the system. One of these universal structures is *asymptotic symmetries*. Generic spacetimes cannot be expected to be endowed with symmetries. In the case of an isolated system in GR, however, intuition suggests that as one recedes from the sources the geometry should become more and more Minkowski-like. This intuition, in turn, makes the notion of asymptotic symmetries plausible. The big surprise is that, in GR, the asymptotic symmetry group does not coincide with the 10-dimensional Poincaré group, but turns out to be an infinite-dimensional group, now called the Bondi–Metzner–Sachs (BMS) group, containing the Lorentz group and a distorted infinite-dimensional group of translations. As pointed out in Penrose’s [27] contribution to this volume, the BMS group first arose from considering the group of transformations which preserve the form of the line elements used in the work of Bondi and collaborators [23,24]. More geometric descriptions were found in subsequent analyses [3,41]. In this spirit, Borthwick & Herfray’s [42] contribution to this volume extends the classical analysis of asymptotic symmetries in asymptotically flat spacetimes to more general classes of spacetimes. This work makes use of promising geometric notions and techniques (projective compactifications and tractor calculus) which are yet to permeate and make an impact in the analytic study of Einstein’s equations, as the conformal technique has done.

In recent years, the study of the BMS group has acquired renewed interest. This has to do, to some extent, with the work by e.g. Hawking, Perry and Strominger on soft hair for black holes and its implications for the information loss paradox [43]. The asymptotic symmetries defined by the BMS group allow the construction of an infinite number of *asymptotic charges*—not all of these conserved but nevertheless satisfying balance laws between cuts of null infinity. These charges can be constructed at both past and future null infinity. A question of particular relevance in the analysis of Hawking *et al*. [43] is the way the past and future asymptotic charges are related: the past and future asymptotic charges can be identified if an *antipodal matching* at spatial infinity is assumed. Friedrich’s framework of spatial infinity (see §4) provides a powerful tool to analyse the genericity of the antipodal identification of past and future null infinity and to characterize the associated class of Cauchy initial data for linear and full nonlinear GR in which this occurs. An overview of this research programme is given in Mohamed’s [44] contribution to this volume. A key outcome of this analysis is that the BMS charges are generically not well-defined unless certain regularity conditions on the initial data are assumed. When they are well defined, however, by writing the charges at the endpoints of null infinity in terms of Cauchy initial data it is possible to recover the identification of past and future charges without the need for an *a priori* assumption of antipodal matching. This analysis suggests that the antipodal identification condition is, at its core, an assumption about the regularity of spatial infinity.

## 4. Friedrich’s conformal Einstein equations and spatial infinity

The Einstein field equations are not conformally invariant. In fact, a naive rewriting of the equations in terms of an unphysical metric $g_{ab}$ conformally related to a physical metric ${\tilde{g}}_{ab}$ yields equations which are formally singular at the conformal boundary. Nevertheless, it was shown by Friedrich [45,46] that there exists a larger system of PDEs, the so-called conformal Einstein field equations, which admit Einstein’s equations as a subsystem and for which standard methods of the theory of partial differential equations are applicable even at the conformal boundary. This observation opened the door to the first proofs of the global existence and nonlinear stability of de Sitter-like and Minkowski-like solutions to the Einstein field equations mentioned earlier [12]. The main idea behind these proofs is that Penrose’s compactification procedure allows one to reformulate an infinite-time existence problem as one where existence only needs to be shown for a finite amount of conformal time. In many relevant problems, it is possible to find a gauge in which the conformal Einstein field equations admit a reduction to a symmetric hyperbolic system. When this is the case, the required existence results follow from the property of Cauchy stability.^1^

Friedrich’s original analysis of the stability of the de Sitter spacetime has been generalized in many directions. In particular, it has allowed the investigation of the global existence of cosmological solutions with spatial sections of constant negative (in the conventions of Friedrich) curvature, see e.g. Minucci’s [48] contribution to this volume. Further, while Friedrich’s analysis was restricted to the vacuum case, there exist now extensions of this strategy to settings involving non-vanishing mass with trace-free energy momentum tensor, as explained in the chapter of Tod [49]. Finally, extensions to the more challenging case where the trace of the energy-momentum is non-vanishing are explored in Friedrich’s [50] article in this volume.

A peculiarity of Penrose’s notion of asymptotic simplicity is that it makes no reference to spatial infinity. In the original paper [2] introducing the conformal method, Penrose himself observed that the presence of a non-vanishing ADM mass in the spacetime gives rise to a singularity of the conformal structure at the point representing spatial infinity. In the following decades, a significant amount of effort was put into trying to understand the relationship between this singular behaviour at spatial infinity and the properties of null infinity (e.g. [3]). These efforts led to the identification by Ashtekar and Hansen of minimal regularity assumptions which allow a matching between spatial and null infinity [7]. As in the case of Penrose’s asymptotically simple spacetimes, the complete classification of Ashtekar and Hansen’s class of AEFANSI spacetimes remains an open question.

As mentioned in §2c, Hintz and Vasy’s proof of the nonlinear stability of Minkowski space, which uses Melrose’s techniques of geometric scattering (see §5), establishes the generic presence of polyhomogeneous asymptotics. There are clear connections between the set-up of Hintz–Vasy and that of Friedrich, and bringing these to the fore, perhaps in application to a full classification of asymptotics of spacetimes, would seem to be a promising area of research.

## 5. Scattering theory

The functional analytic framework of the Lax–Phillips scattering theory [51] for hyperbolic differential equations, developed in the 1960s, provides a point of departure for scattering in general relativity. Lax and Phillips deal with systems described by a group of unitary operators ${\left\{U(t)\right\}}_{t}$ acting on a Hilbert space H which possesses distinguished subspaces $D_{-}$ and $D_{+}$ [52]. These subspaces, to be interpreted as spaces of scattering data in the past and future, have the property that $U(t)D_{-}$ increases monotonically from the zero subspace to the whole space H as t varies from −∞ to ∞, and analogously for $U(t)D_{+}$, which decreases from H to zero as t varies from −∞ to ∞. With each of $D_{\pm }$, there is associated a particular spectral representation of ${\left\{U(t)\right\}}_{t}$, and the future and past representations are related by a unitary operator S, the scattering matrix.

Each spectral representer of the solution can be understood as a so-called asymptotic profile (i.e. the leading-order behaviour of the solution near null infinity) in the past and the future. In the early 1980s, Friedlander [53] reinterpreted the Lax–Phillips asymptotic profiles as his radiation fields [54–56], i.e. limits along null geodesics of the scattered fields multiplied by a radial coordinate r. The radiation fields had been known to be simply the restrictions of the conformally rescaled fields to I in Penrose’s conformal compactification. Friedlander was therefore able to give the first construction of a ‘conformal’ scattering theory for the free wave equation. In Friedlander’s picture, the property that the Lax–Phillips asymptotic profiles completely determine the solution in the interior of the spacetime became the problem of solving a characteristic Cauchy problem from I. It is here that Friedlander opened the way for a geometric formulation of scattering: while the Lax–Phillips theory, being based on the use of spectral methods, was not applicable on time-dependent backgrounds, Friedlander’s picture clarified that this ought to be understood as a technical shortcoming, the relevant information for scattering being contained in the asymptotics of the physical fields. Moreover, for massless fields these asymptotics were elegantly accessible via Penrose’s conformal compactification. The combination of these ideas has become known as conformal scattering [8]. A detailed comparison of the spectral and conformal approaches to scattering, and the difficulties and advantages typically seen in each approach, is given in Nicolas’s [57] contribution to this volume.

A related major development in the field of mathematical relativity has been the realization of the close connections between the ideas behind Penrose’s compactification and Melrose’s school of microlocal analysis, often called *geometric scattering* [58]. Very briefly, geometric scattering attempts to develop a Fredholm theory for hyperbolic operators on appropriately (but not necessarily conformally) compactified manifolds. The methods of geometric scattering have proven a powerful tool to analyse the nonlinear stability and asymptotics of solutions to the Einstein field equations, including black hole solutions [59,60]. Applications of the techniques of geometric scattering to the study of asymptotics in general relativity are discussed in Hintz’s [61] contribution to this volume, where asymptotically de Sitter-like spacetimes are constructed from asymptotic data (prescribed at the conformal boundary). This result generalizes the classical proof by Friedrich [12] (which is restricted to four dimensions) and that of Anderson [62] (which only applies to Lorentzian manifolds with odd spatial dimensions).

## 6. Black holes

The development of a complete and satisfactory mathematical theory of black holes has long been one of the main aims of mathematical relativity. This subject has been made even more relevant by the direct experimental observation in 2015 of gravitational waves produced by the coalescence of a binary system of black holes [63]. From the mathematical side, a gargantuan effort has been poured into developing a proof of the nonlinear stability of the Kerr spacetime [64–66].

The notion of null infinity as describing an idealized far away observer is central to the classical definition of a black hole in the asymptotically flat setting [67,68]. While there are very precise conjectures (and proofs under certain not entirely satisfactory assumptions) regarding the uniqueness of stationary black holes, the question of the uniqueness in the cosmological setting is not well understood [69]. Conformal methods allow the study of the uniqueness of black holes in spacetimes with a positive cosmological constant by means of an asymptotic initial value problem in which initial data are prescribed at the (spacelike) conformal boundary. The contribution of Mars & Peón-Nieto [70] to this volume describes an ongoing research programme to understand, from the point of view of asymptotic initial data, the sense in which the Kerr–de Sitter family of solutions is special. This programme should provide important insights into the question of the uniqueness of stationary black holes in the cosmological setting.

One of the most promising sources of gravitational waves for the future generation of space-based detectors (LISA) are the so-called extreme mass-ratio inspirals (EMRI) [71]. These are black hole binary systems in which one of the components can be treated as a point source and the gravitational waves produced can be adequately treated by means of perturbation theory [72]. As EMRIs produce a gravitational wave signal over extended periods of time, the production of wave templates to be used in their detection require very precise numerical simulations. The use of hyperboloidal foliations to numerically construct the solutions to the equations of perturbation theory provides a way of constructing the signal templates which avoids the use of problematic boundary conditions. The key idea is that null infinity provides a natural *outflow boundary* on which no boundary conditions need to be prescribed. This promising application of conformal methods to the study of gravitational waves is described in Panosso Macedo’s [73] contribution to this volume.

## 7. Future directions

We hope that the contributions to the present volume convey to the reader the influence in current research of Penrose’s idea of studying the asymptotics of fields in general relativity using conformal geometry. To conclude, we would like to point out two directions of research which appear to us to be quite promising.

The first is to further explore and develop the connections between the methods of geometric scattering and the perhaps more traditional uses of conformal methods in general relativity, e.g. using Friedrich’s conformal Einstein field equations. It is possible that a combination of the two perspectives could yield a fruitful approach to a sharp classification of decay rates of initial data and the asymptotic properties of the resulting spacetimes. In a similar vein, it would be interesting to investigate if questions of nonlinear stability of black holes might be addressed using the conformal Einstein field equations, probably in some conjunction with the ideas that are already well established within the black hole stability community.

The second direction is to continue the development of numerical schemes to construct solutions to the Einstein field equations in a conformal setting: see e.g. the work by Hübner on the numerical implementation of the hyperboloidal initial value problem [74]. The value of these numerical simulations should not be underestimated and can provide valuable insights for further analytical studies. In particular, the use of conformal methods should pave the way to the numerical construction of complete scattering spacetimes which are prescribed through initial data on the whole of past null infinity—this numerical endeavour will require input from the analytic side, as described in the previous paragraph.

## Data Availability

This article has no additional data.

## Footnotes

1 A similar strategy was used by Y. Choquet-Bruhat and D. Christodoulou to show the global existence of solutions to the Yang–Mills equations [47].
